# Extension of the PRISMA 2020 statement for living systematic reviews (LSRs): protocol

**DOI:** 10.12688/f1000research.75449.2

**Published:** 2022-06-10

**Authors:** Lara A Kahale, Vanessa Piechotta, Joanne E McKenzie, Elena Dorando, Claire Iannizzi, James M Barker, Matthew J Page, Nicole Skoetz, Elie A Akl

**Affiliations:** 1 Cochrane Central Executive, Cochrane, London, UK; 2 Department I of Internal Medicine, Center for Integrated Oncology Aachen Bonn Cologne Duesseldorf, Cologne, Germany; 3 Faculty of Medicine and University Hospital Cologne, University of Cologne, Cologne, Germany; 4 School of Public Health and Preventive Medicine, Monash University, Melbourne, Australia; 5 F1000 Research, London, UK; 6 Department of Internal Medicine, American University of Beirut, Beirut, Lebanon

**Keywords:** PRISMA, extension, living systematic reviews, reporting, flow diagram, checklist, statement

## Abstract

**Background**: While the PRISMA 2020 statement is intended to guide the reporting of original systematic reviews, updated systematic reviews, and living systematic reviews (LSRs), its explanation and elaboration document notes that additional considerations for updated systematic reviews and LSRs may need to be addressed. This paper reports the protocol for developing an extension of the PRISMA 2020 statement for LSRs.

**Methods:** We will follow the EQUATOR Network’s guidance for developing health research reporting guidelines. We will review the literature to identify possible items of the PRISMA 2020 checklist that need modification, as well as new items that need to be added. Then, we will survey representatives of different stakeholder groups for their views on the proposed modifications of the PRISMA 2020 checklist. We will summarize, present, and discuss the results of the survey in an online meeting, aiming to reach consensus on the content of the LSR extension. We will then draft the checklist, explanation and elaboration for each item, and flow diagram for the PRISMA 2020 extension. Then, we will share these initial documents with stakeholder representatives for final feedback and approval.

**Discussion**: We anticipate that the PRISMA 2020 extension for LSRs will benefit LSR authors, editors, and peer reviewers of LSRs, as well as different users of LSRs, including guideline developers, policy makers, healthcare providers, patients, and other stakeholders.

## Introduction

Systematic reviews (SRs) need to include all relevant primary studies to validly answer clinical or public health questions. To meet this goal, SRs need to be updated on a frequent basis, particularly in areas with a fast pace of research generation and publication (e.g., treatments for COVID-19). Living systematic reviews (LSRs) attempt to achieve this through continual searching of the literature and incorporation of relevant new evidence, soon after it becomes available. ^1^ In addition to the frequency of the search update, LSRs differ from traditional SRs in a number of other aspects, e.g., reporting on the change in eligibility criteria in results, conclusion, authorship, and the publication process. Also, unlike traditional SRs, LSRs should consider how to avoid inadvertent type I and II errors arising from repeated updating. It is not clear whether LSRs are reporting on how they have addressed this issue.

The ‘Preferred Reporting Items for Systematic reviews and Meta-Analyses’ (PRISMA) statement is widely used to report SRs. ^2^ The statement is intended to facilitate transparent, complete, and accurate reporting of SRs. While the PRISMA 2020 statement is intended to guide the reporting of original systematic reviews, updated systematic reviews, and LSRs, its explanation and elaboration document notes that additional considerations for updated and LSRs may need to be addressed. ^3^

A recent methodological survey of LSRs identified variability in reporting the flow of studies through the different phases of the review for the different update versions. ^4^ Another mixed-methods study found that authors of LSRs have adopted different approaches to communicating the LSR findings to readers. ^5^ One of the stated reasons for variability was that little or no guidance exists as to which approach is best for reporting LSRs. At the moment, there appears to be a lot of variation in how LSRs are reported. An extension to the current PRISMA 2020 statement is needed to better address the unique aspects of LSR reporting.

### Objective

The objective of this study is to develop an extension of the PRISMA 2020 statement for LSRs. The aim of this paper is to report the details of the protocol for that study. We adopt the definition of a LSR as being “a systematic review that is continually updated, incorporating relevant new evidence as it becomes available”. ^1^

## Protocol

**Figure 1.**
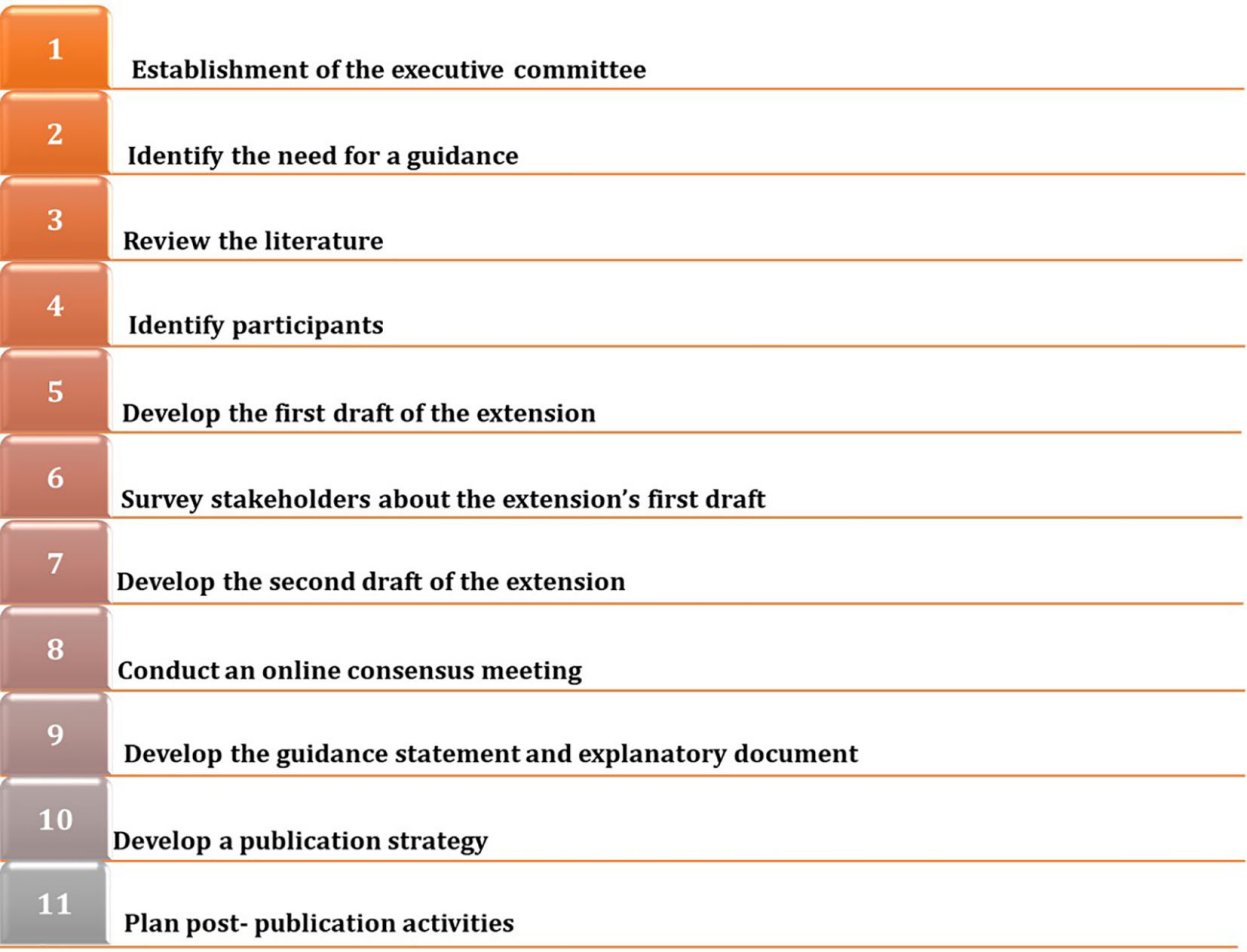
Steps for the development of an extension to the PRISMA 2020 statement for living systematic reviews.

We will develop an extension to the PRISMA 2020 statement for LSRs by following the EQUATOR Network’s guidance for developing health research reporting guidelines ( Figure 1). ^6^ We have registered our project on the EQUATOR Network website. ^7^ We will obtain ethical approval for the study and will inform participants that any collected information will be de-identified and that findings will be reported in aggregate.

### Initial steps

*Establishment of the executive committee*

The executive committee members, who are the authors of this protocol, will lead, organize, and author this extension. The executive committee consists of individuals with various backgrounds, including systematic review methodologists, LSR authors, co-leads and authors of the PRISMA 2020 statement, publishers, clinicians, and practice guideline methodologists.

*Identify the need for a guidance*

As noted earlier, there are a number of aspects of LSRs that the guidance from the PRISMA 2020 statement does not address, or where we believe modification is needed. Furthermore, we are not aware of any reporting guidance for LSRs published outside of the PRISMA banner.

*Review the literature*

To identify possible items of the PRISMA 2020 checklist that need to be added or modified for the reporting of LSRs, we will make use of the cumulative information arising from five methodological studies examining aspects of LSRs, and led by members of the executive committee:

• *Methodological survey of published LSR flow diagrams* ^4^
  This survey examined how authors of all LSRs published since 2014 until April 2021 reported the flow of studies through the different phases of the review for each update. We noted six different ways of reporting study flow, none of which are currently required in the PRISMA 2020 flow diagram.
• *Concept paper on methodological challenges with COVID-19 LSRs* ^8^
  This concept paper reviews the methodological challenges of conducting LSRs during the COVID-19 pandemic. The paper identified several reporting challenges including, for example, how to transparently report methodological changes when updating an LSR. We will consider how to address these challenges in the extension of the PRISMA 2020 checklist.
• *Methodological survey of published LSRs* ^9^
  This survey of published LSRs (published between 2014 until April 2021) describes their general characteristics, the methods of conduct and reporting, and how the methods change across updates. Also, it examined how and when the different versions of an LSR were being published. The practice of authors aids in identifying relevant items that may not have been addressed in the PRISMA 2020 checklist.
• *Scoping review of the methodological literature on LSRs* ^10^
  This scoping review examines methodological guidance for conducting, reporting, and publishing LSRs (protocol available ^10^). Results from this review will be used to identify issues pertinent to the reporting of LSRs. These issues will be mapped against the 27 items of the PRISMA 2020 checklist, thus allowing identification of items that need modification, and the items that need to be added.
• *Qualitative study with LSR authors*
  We will interview LSR authors and journal editors to explore their views and their experiences with conducting, reporting, and publishing LSRs. We will use the findings to identify potential modifications to the PRISMA 2020 checklist items.

### Pre-meeting activities

*Identify participants*

We will identify representatives of different stakeholders groups, including the authors of PRISMA 2020; authors of published PRISMA extensions ^11^^–^^18^; editors of journals that have published LSRs; representatives of organizations that have published LSRs (e.g., Cochrane, Campbell); publishers and editors of preprint platforms; practice guideline developers (e.g., the World Health Organization); practice guideline end-users (e.g., health care professionals, patients); end-users of the PRISMA LSR extension (i.e., LSR authors); systematic review methodologists; statisticians; information specialists; science communication experts; programmers for large databases and systematic review software; and research funders.

We will invite one to three representatives for each type of stakeholder (listed above). We will aim to have adequate gender and geographic representation.

*Develop the first draft of the extension*

We will develop the first draft of the checklist extension based on the results of the background studies (see ‘Review of the literature’ section). Then, the executive committee members will complete a survey to propose the items of the 2020 PRISMA checklist that may be modified. They will also propose new items that may be added.

*Survey stakeholders about the extension’s first draft*

In preparation for this step, we will summarize the findings of the background studies (see ‘review of the literature’ section) and share them with stakeholders. Then, we will survey the stakeholders for their views on each of the items included in the draft extension of the PRISMA 2020 checklist. For each item currently included in the PRISMA 2020 checklist, we will present its wording, its explanation and elaboration, and possibly a suggestion on how the item may be modified for LSRs. Then, for each item, we will seek stakeholders’ views on whether the item should be kept without changes or modified. We will ask similar questions for new items proposed by the executive committee. In addition, we will ask stakeholders to propose additional items to include in the checklist. We will calculate descriptive statistics using frequencies and percentages of responses to each item.

*Develop the second draft of the extension*

We will use the results of the survey to develop a second draft of the extension. Similar to the development process used for the PRISMA 2020 checklist, we will consider that an item has reached consensus when one of its response options is selected by more than 66% of the stakeholders. ^19^ Items for which no consensus is reached will be considered for discussion at a consensus meeting.

### Meeting

*Conduct an online consensus meeting*

We will conduct an online meeting to try and reach consensus on the content of the LSR extension. We will electronically share the second draft of the extension ahead of the meeting. The meeting will be held via an online meeting platform with enough sessions to cover the tasks to be completed and to accommodate the different time zones of those attending. Members of the executive team will chair the meeting and take minutes.

During the consensus meeting, we will give short presentations on the background, rationale, the scope of the project, the results of the survey. We will discuss the proposed list of modifications to the checklist (any new items and modifications of existing items of the PRISMA 2020 statement), and to the PRISMA flow diagram.

We will prioritize items for discussion for which there is a lack of consensus. The discussion will revolve around the different arguments for inclusion or exclusion of a specific item with the aim of reaching consensus. If outstanding issues are not resolved (not discussed, consensus not reached), we will re-work them post-meeting and share them via email with the meeting attendees for their comments.

If time allows, we will discuss the strategy for writing the manuscript, task assignment, authorship, and ideas regarding piloting and knowledge translation.

### Post-meeting

*Develop the guidance statement and explanatory document*

Members of the executive committee will draft initial documents including (1) the modified PRISMA 2020 checklist for LSRs, (2) explanation and elaboration for each item, and (3) modified flow diagram. These initial documents will then be circulated to all members of the executive committee and to all meeting attendees for feedback and approval. We will seek their views on the layout, clarity of the terminology, and the comprehensiveness of the set of items covered. Any proposed revision arising from these comments will be assessed by the executive committee as to whether further changes are required, including lumping and splitting.

*Develop a publication strategy*

We will publish the extension in an open-access, peer reviewed journal and upload a pre-print and the material which informed the extension (e.g., de-identified survey data) to the Open Science Framework repository.

### Post publication activities

*Plan post- publication activities*

We will post this extension on the PRISMA statement website and will develop an application that facilitates the development/creation of a PRISMA 2020 flow diagram for LSRs. We will encourage readers and users to submit any comments or feedback via the website.

We will welcome and support any initiative to translate the extension or any part of it to a different language. We will contact editors of journals that publish LSRs to inform them about the extension and to seek their endorsement. Specifically, we will encourage journal editors and publishers to raise awareness of the extension by referring to it in journal “Instructions to authors”, endorsing its use, advising editors and peer reviewers to evaluate the reporting of submitted LSRs against this extension, and making changes to journal policies to accommodate the extension.

### Study status

Currently, we are reviewing the literature, identifying the participants, and developing the first draft of the extension.

## Discussion

### Summary

The number of LSRs has substantially increased over time, and this is set to keep growing. Complete and accurate reporting of the methods and results of these reviews is necessary for readers to understand the findings, and identify any methodological weaknesses, that may compromise those findings. In this protocol we have outlined the steps we will take to develop a reporting extension to PRISMA 2020 specific to LSRs.

### Strengths and limitations

Conducting online meetings to try reach consensus might have some advantages and challenges. An advantage is that such meetings are more feasible and convenient for attendees with busy schedules and ensures equity, especially for those who do not have funding to attend in-person meetings. On the other hand, there are time zone challenges when participants are attending from around the globe. To address this, we will try to accommodate the attendees’ different time zones. A further challenge relates to technical issues associated with Internet connection or other software failures. To address these challenges, we will ask the attendees to secure an alternative to the primary Internet connection in case the latter fails. A further strength is that members of the executive committee have diverse backgrounds and expertise at all levels that will enrich and facilitate completion of the extension.

### Implication for practice

We anticipate that the PRISMA 2020 extension for LSRs will benefit LSR authors, editors, and peer reviewers of LSRs, and different users of LSRs, including guideline developers, policy makers, healthcare providers, patients, and other stakeholders. We hope that implementation of the reporting guidance will lead to more transparent, complete, and accurate accounts of LSRs, thus providing the necessary synthesized evidence to underpin healthcare decisions.

## Data availability

### Underlying data

No data are associated with this article.

## References

[1] Elliott JH : Living systematic review: 1. Introduction-the why, what, when, and how. *J. Clin. Epidemiol.* 2017;91:23–30. 10.1016/j.jclinepi.2017.08.010 28912002

[2] Page MJ Moher D : Evaluations of the uptake and impact of the Preferred Reporting Items for Systematic reviews and Meta-Analyses (PRISMA) Statement and extensions: a scoping review. *Syst. Rev.* 2017;6(1):263. 10.1186/s13643-017-0663-8 29258593 PMC5738221

[3] Page MJ : PRISMA 2020 explanation and elaboration: updated guidance and exemplars for reporting systematic reviews. *BMJ.* 2021;372:n160. 10.1136/bmj.n160 33781993 PMC8005925

[4] Kahale LA : Tailored PRISMA flow diagrams for living systematic reviews: a methodological survey and a proposal. *F1000Res.* 2021;10(192):192. 10.12688/f1000research.51723.1 35136567 PMC8804909

[5] Millard T : Feasibility and acceptability of living systematic reviews: results from a mixed-methods evaluation. *Syst. Rev.* 2019;8(1):1–14. 10.1186/s13643-019-1248-5 31837703 PMC6911272

[6] Moher D : Guidance for developers of health research reporting guidelines. *PLoS Med.* 2010;7(2):e1000217. 10.1371/journal.pmed.1000217 20169112 PMC2821895

[7] Equator Network: *PRISMA for LSR – Extension of PRISMA 2020 for living systematic reviews.* 2021. Reference Source

[8] Iannizzi C : Methodological challenges for living systematic reviews conducted during the COVID-19 pandemic: a concept paper. *J. Clin. Epidemiol.* 2022;141(82-9):82–89. 10.1016/j.jclinepi.2021.09.013 34525406 PMC8435072

[9] Khamis AM : Methods of conduct and reporting of living systematic reviews: a protocol for a living methodological survey. *F1000Res.* 2019;8:221. 10.12688/f1000research.18005.1 31231512 PMC6556985

[10] Iannizzi C : Methods and guidance on conducting, reporting, publishing and appraising living systematic reviews: a scoping review protocol. *F1000Res.* 2021;10(802):802. 10.12688/f1000research.55108.1 35186269 PMC8822136

[11] Welch V : PRISMA-Equity 2012 extension: reporting guidelines for systematic reviews with a focus on health equity. *PLoS Med.* 2012;9(10):e1001333. 10.1371/journal.pmed.1001333 23222917 PMC3484052

[12] Beller EM : PRISMA for Abstracts: reporting systematic reviews in journal and conference abstracts. *PLoS Med.* 2013;10(4):e1001419. 10.1371/journal.pmed.1001419 23585737 PMC3621753

[13] Hutton B : The PRISMA extension statement for reporting of systematic reviews incorporating network meta-analyses of health care interventions: checklist and explanations. *Ann. Intern. Med.* 2015;162(11):777–784. 10.7326/M14-2385 26030634

[14] Stewart LA : Preferred Reporting Items for Systematic Review and Meta-Analyses of individual participant data: the PRISMA-IPD Statement. *JAMA.* 2015;313(16):1657–1665. 10.1001/jama.2015.3656 25919529

[15] Zorzela L : PRISMA harms checklist: improving harms reporting in systematic reviews. *BMJ.* 2016;352:i157. 10.1136/bmj.i157 26830668

[16] Guise JM : AHRQ series on complex intervention systematic reviews-paper 6: PRISMA-CI extension statement and checklist. *J. Clin. Epidemiol.* 2017;90:43–50. 10.1016/j.jclinepi.2017.06.016 28720516

[17] McInnes MDF : Preferred Reporting Items for a Systematic Review and Meta-analysis of Diagnostic Test Accuracy Studies: The PRISMA-DTA Statement. *JAMA.* 2018;319(4):388–396. 10.1001/jama.2017.19163 29362800

[18] Tricco AC : PRISMA Extension for Scoping Reviews (PRISMA-ScR): Checklist and Explanation. *Ann. Intern. Med.* 2018;169(7):467–473. 10.7326/M18-0850 30178033

[19] Page MJ : Updating guidance for reporting systematic reviews: development of the PRISMA 2020 statement. *J. Clin. Epidemiol.* 2021;134:103–112. 10.1016/j.jclinepi.2021.02.003 33577987

