# Supplementary material for: Extension of the PRISMA 2020 statement for living systematic reviews (LSRs): protocol
Source: F1000Res. 2022 Jun 10;11:109. Originally published 2022 Jan 28. [Version 2] doi: 10.12688/f1000research.75449.2 (PMC11134135; doi:10.12688/f1000research.75449.2)
Supplement: Peer review history

# Reviewer report: Reviewer response for version 1

**DOI:** 10.5256/f1000research.79324.r121603

**Published:** 2024-05-28

**Authors:** Zhao-Xiang Bian, Yuting Duan

**Affiliations:** 1 Chinese EQUATOR centre, Hong Kong Baptist University, Hong Kong SAR, China; 2 Chinese EQUATOR centre, Hong Kong Baptist University, Hong Kong SAR, China, Hong Kong Baptist University, Hong Kong SAR, China

The protocol for developing the extension of Living systematic reviews to PRISMA 2020 is a good one. The topic is important during pandemics such as COVID-19. Some suggestions are as follows:

1. Provide the detailed methods to the survey for the stakeholders. The survey process is crucial to get the consensus of the reporting guideline. Currently, the methods to decide whether the items are included, excluded, or discussed at the meeting are not clear.
2. Add the pilot test after the consensus meeting. The pilot testing will provide practical feedback for the use of the checklist.

Is the study design appropriate for the research question?

Yes

Is the rationale for, and objectives of, the study clearly described?

Yes

Are sufficient details of the methods provided to allow replication by others?

Partly

Are the datasets clearly presented in a useable and accessible format?

Yes

Reviewer Expertise:

Reporting guideline methodology; Clinical Trial Design and implementation; Evidence-based Medicine.

We confirm that we have read this submission and believe that we have an appropriate level of expertise to confirm that it is of an acceptable scientific standard.

# Reviewer report: Reviewer response for version 1

**DOI:** 10.5256/f1000research.79324.r125876

**Published:** 2022-04-06

**Authors:** Meera Viswanathan

**Affiliations:** 1 RTI International, Research Triangle Park, NC, USA

I have a few suggestions for improvement; please use or discard as you like.

 It may be that you intend on doing this when you present the results of your scoping review, but a first step that I didn’t see explicitly was ensuring that all your participants are working from a common definition of a living systematic review. You’ll be able to come together more easily on required PRISMA items if all participants share a common conceptual understanding of the boundaries between living systematic reviews and frequently updated systematic reviews. Do all participants share a common understanding of how much change in core methods or scope is permissible?

 I also think you might benefit from including two more groups: (1) programmers for large databases and systematic review software and (2) science communication experts. I suggest the former group because large databases are changing methods. NLM, for example, is switching to automated indexing ( MEDLINE 2022 Initiative: Transition to Automated Indexing. NLM Technical Bulletin. 2021 Nov–Dec (nih.gov)); these changes may have implications for how searches should be reported in LSRs. Similarly, systematic review software (and AI tools within them) is likely to play a significant role in LSRs, and these software developers may have insights on upcoming technological changes with implications for reporting. As for the latter group, I think it would be interesting to learn what types of reporting enhance trust in the findings of LSRs, when results are updated (and perhaps prior errors are corrected) on an ongoing basis.

 Finally, I think that even after the consensus meeting, you may have to do some lumping and splitting, and it’s best to anticipate that activity and be transparent about it.

Is the study design appropriate for the research question?

Yes

Is the rationale for, and objectives of, the study clearly described?

Yes

Are sufficient details of the methods provided to allow replication by others?

Partly

Are the datasets clearly presented in a useable and accessible format?

Not applicable

Reviewer Expertise:

Systematic review methodologist

I confirm that I have read this submission and believe that I have an appropriate level of expertise to confirm that it is of an acceptable scientific standard.

# Response

**Published:** 2022-06-01

**Authors:** Lara Kahale

**Affiliations:** American University of Beirut, Lebanon

The Reviewers’ comments are in **bold** font and our replies in regular font. Extracts from the text are in *italic* fonts with changes *underlined*. We have indicated the sections where revisions have been made in our manuscript.

**Reviewer 1: Meera Viswanathan, RTI International, Research Triangle Park, NC, USA**

**I have a few suggestions for improvement; please use or discard as you like.**

**1. It may be that you intend on doing this when you present the results of your scoping review, but a first step that I didn’t see explicitly was ensuring that all your participants are working from a common definition of a living systematic review. You’ll be able to come together more easily on required PRISMA items if all participants share a common conceptual understanding of the boundaries between living systematic reviews and frequently updated systematic reviews. Do all participants share a common understanding of how much change in core methods or scope is permissible?**

- Authors' response: Thank you so much for your suggestion. We have added the following to our objectives section: *“We adopt the definition of an LSR as being “a systematic review that is continually updated, incorporating relevant new evidence as it becomes available”.*

**2. I also think you might benefit from including two more groups: (1) programmers for large databases and systematic review software a nd (2) science communication experts. I suggest the former group because large databases are changing methods. NLM, for example, is switching to automated indexing (MEDLINE 2022 Initiative: Transition to Automated Indexing. NLM Technical Bulletin. 2021 Nov–Dec (nih.gov)); these changes may have implications for how searches should be reported in LSRs. Similarly, systematic review software (and AI tools within them) is likely to play a significant role in LSRs, and these software developers may have insights on upcoming technological changes with implications for reporting. As for the latter group, I think it would be interesting to learn what types of reporting enhance trust in the findings of LSRs, when results are updated (and perhaps prior errors are corrected) on an ongoing basis.**

- Authors' response: Per your suggestion, we have now invited programmers for large databases and systematic review software. We had already invited science communication experts and have invited one more.

**3. Finally, I think that even after the consensus meeting, you may have to do some lumping and splitting, and it’s best to anticipate that activity and be transparent about it.**

- Authors response:  thank you. We have added text referring to that point: *‘Any proposed revision arising from these comments will be assessed by the executive committee as to whether further changes are required* *, including lumping and splitting’.*
